# Phenotype correlation analysis and excellent germplasm screening of herb *Bletilla* Rchb. f. based on comprehensive evaluation from thirty-three geographic populations

**DOI:** 10.1186/s12870-022-03540-w

**Published:** 2022-03-29

**Authors:** Junfeng Huang, Fang Yuan, Ming Zhou, Tianyue Huang, Yanjun Zhang, Qiong Liang

**Affiliations:** Key Laboratory of Plant Germplasm Enhancement and Specialty Agriculture, Wuhan Botanical Garden, Chinese Academy of Sciences, Wuhan, 430074 China

**Keywords:** Active ingredients, *Bletilla*, Correlation analysis, Gray relation analysis, Phenotypic traits

## Abstract

**Background:**

The *Bletilla* genus of Orchidaceae includes plants with great economic value, among which *B. striata* is the main traditional medicinal plant, and its pseudobulb, known as *BaiJi*, was first recorded in Shennong’s Classic of Materia Medica. However, there has been little systemic evaluation of the germplasm quality of *Bletilla* plants in China. In order to comprehensive evaluate the *Bletilla* resources in China and screen out the candidate phenotypic traits determining yield and/or quality of *Bletilla*, the variation of phenotypic indicators (pseudobulb, leaf, stem, inflorescence, flower) and active ingredients contents (polysaccharide, total phenolics and militarine) in different populations of *B. striata* and *B. ochracea* were investigated through 4 years’ common-garden experiment.

**Results:**

There were abundant phenotypic variations and significant differences among different populations in the morphological phenotypes, pseudobulb weight and main active ingredient contents. AHBZ, HBLT and HBSN populations showed good prospects for industrial development, presenting higher quality in terms of yield and main active ingredient content. Pseudobulb yield, polysaccharide and total phenol content are positively correlated with phenotypic traits. Militarine content is negatively correlated with almost all indexes. Plant height, leaf width and stem diameter may be important indicators of potential excellent germplasms.

**Conclusions:**

*Bletilla* is not strictly geoauthentic medicinal plants. *B. ochracea* could be accepted as an alternative resource to *B. striata*. The best harvest period of *Bletilla* is the third year after cultivation. Plant height, leaf width and stem diameter may be important indicators of potential excellent germplasms. These results provide important information required for the efficient screening and utilization of *Bletilla* germplasm resources.

**Supplementary Information:**

The online version contains supplementary material available at 10.1186/s12870-022-03540-w.

## Background

*Bletilla* Rchb. f. (Orchidaceae) is a small genus with six species distributed in Asia from northern Myanmar and southern and eastern China to Japan and Korea. China is the distribution center of *Bletilla* with four species: *B. sinensis* (Rolfe) Schltr., *B. formosana* (Hayata) Schltr., *B. striata* (Thunb. ex A. Murray) Rchb. f. and *B. ochracea* Schltr. [1] (http://www.iplant.cn/info/Bletilla?t=foc). The dried pseudobulb of *B. striata* was recorded as *BaiJi* in the Chinese Pharmacopeia, and was first recorded in Shennong’s Classic of Materia Medica [2]. As the main traditional medicinal plant, *B. striatai* is the only original plant, *B. ochracea* and *B. formosana* are also used as local medications or substitutes [3]. Currently, the research of *Bletilla* mainly focuses on chemical components, pharmacological activities and reproductive techniques [4–9].

As the main active ingredient, *B. striata* polysaccharide (BSP) is widely used in clinical hemostasis, and can also be used as an excellent biopolymer material and pharmaceutical excipient [10–12]. Polysaccharide is rich in the dried pseudobulb with content of 20–50% [13, 14]. Moreover, approximately 211 compounds have been extracted from *B. striata* pseudobulbs, including glucosides, bibenzyls, phenanthrenes, quinones, stilbenes, triterpenoids, phenolics etc. [5, 9, 15, 16]. Militarine (1,4-bis [4-(glucooxy) benzyl]-2-isobutyl malate (C_34_H_46_O_17_)) shows the highest content in the nonpolysaccharide fraction in *B. striata* [17], with content ranging from 1.45% to 9.28% [18]. BSP and militarine are suggested quality markers for *B. striata* [3, 19]. Additionally, phenolics are important active compounds in *B. striata*, and the total phenol content varied from 1.83% to 13.73%.

Common garden experiments retaining the same cultural environment can be used to analyze phenotypic variation among different populations to eliminate phenotypic variation induced by environmental factors and have become an important means of resource evaluation [20–22]. Through the collection of wild resources for common garden cultivation, quality evaluation was performed to determine the high-quality resources in the tested varieties and has been widely used in many medicinal plants, such as *Epimedium sagittatum* and *Lycium chinense* [22, 23]. Gray relation analysis (GRA) is a multifactor statistical analysis method that includes dimensionless processing, correlation coefficient calculation, correlation degree and relative correlation degree calculation and is widely used in the comprehensive evaluation of the quality of medicinal materials [24]. The correlation is used in the context of a linear relationship between 2 continuous variables and the Pearson correlation coefficient is typically used for jointly normally distributed data [25]. Correlation analysis can be used to measure the relationship among yield, quality and agronomic traits to determine which trait affects yield and/or quality [26, 27].

Systematic collection of wild and cultivated resources, investigation of phenotypic traits and active ingredient content, and selection of excellent germplasm are an important part of the effective utilization of medicinal plants [3]. Small-scale collection and resource evaluation of *Bletilla* have been performed. For example, Chinese researchers evaluated the germplasm resources of *B. formosana* in Yunnan Province, determined the total phenol content of *B. striata* in northwest of Hubei Province, and determined the polysaccharide content of *B. striata* from different areas in Anhui and Guizhou Provinces, while these studies have focused on phenotypic traits or multiple active components in a small region, or single component analysis in multiple provinces, lacking a large-scale collection and comprehensive evaluation of *Bletilla* resources in China [28]. Therefore, we selected 33 *Bletilla* populations, which covered their major distribution areas in China, to systematically evaluate the main phenotypic traits, pseudobulb yields and main active ingredients among different species and populations. We aimed to screen higher-quality germplasm resources of *Bletilla* and the key indicators related to economic characteristics, which will provide important information for the efficient breeding of *B. striata*. Our study will enhance our ongoing efforts in the sustainable development of traditional medicinal plants.

## Results

### Morphological measurement and resource evaluation of pseudobulb weight

**Fig. 1:**
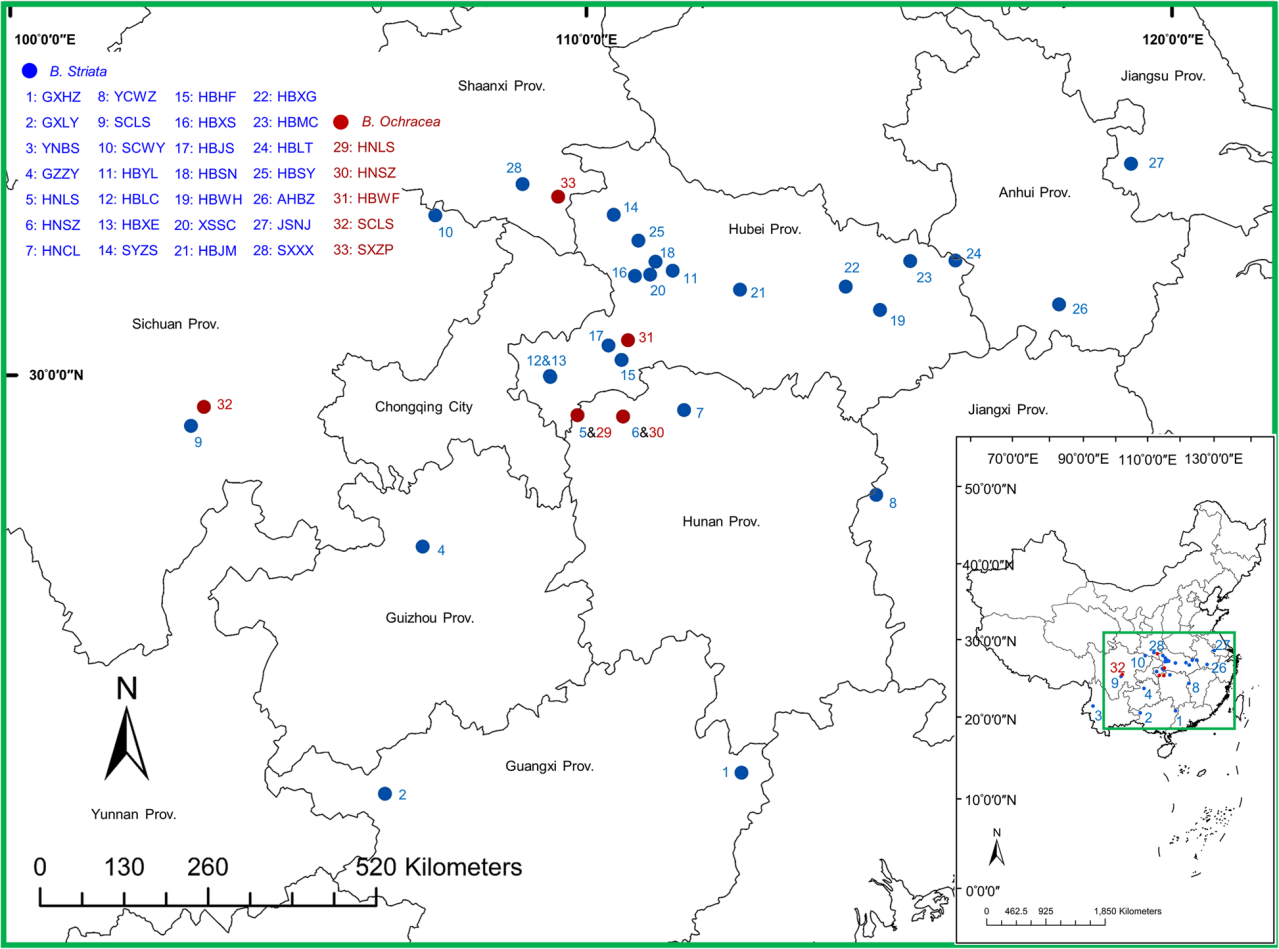
Geographic distribution of the *Bletilla* plants collected from China. The 33 populations of *Bletilla* across ten provinces in China. For location details, see Table S1. This map was drawn by ArcGIS software based on longitude and latitude in population information (Table S1). No. 1–28 are populations of *B. striata* (in blue), while No. 29–33 are populations of *B. ochracea* (in red)

**Fig. 2:**
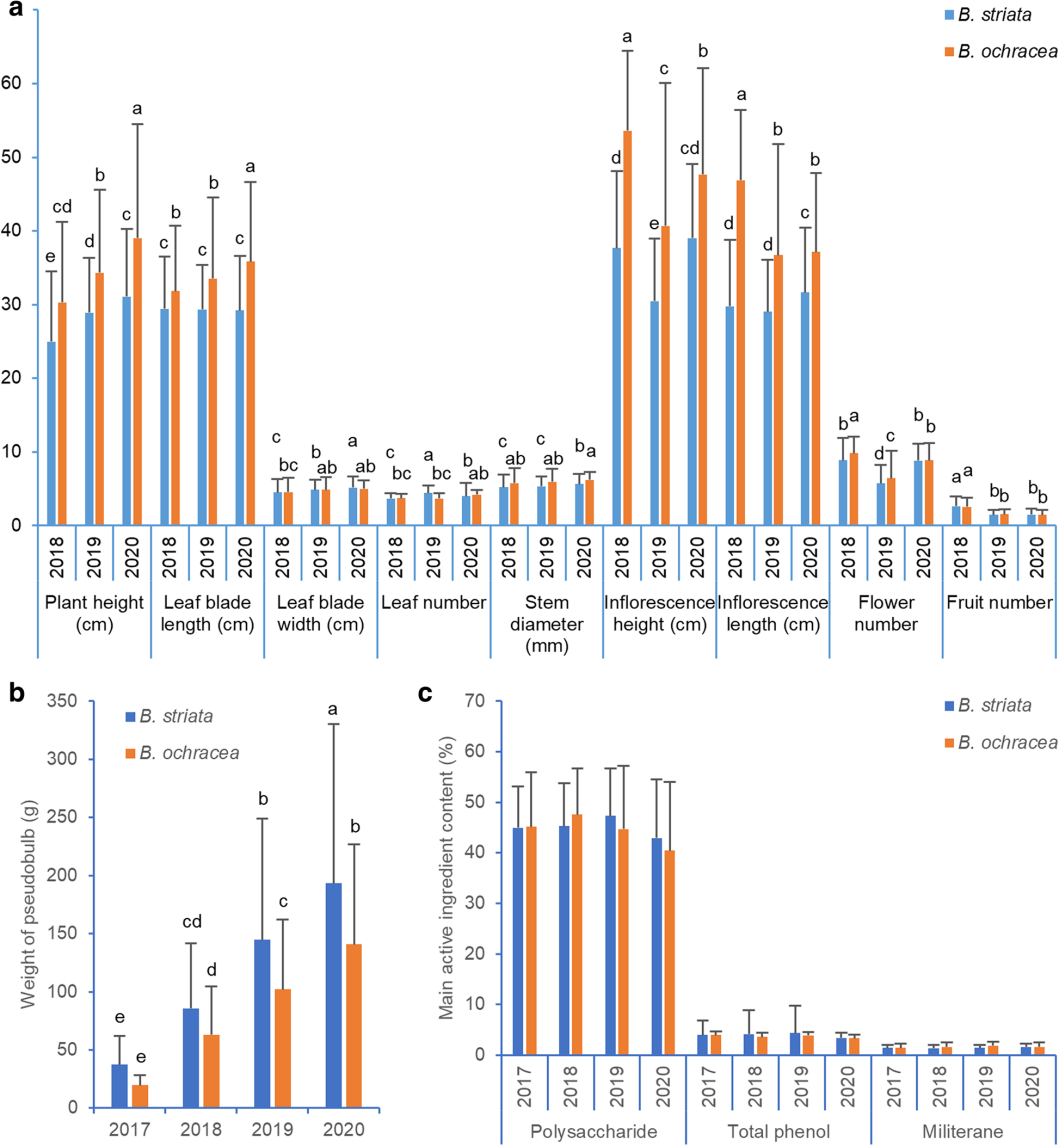
Summary of morphological measurements, pseudobulb weight and main active ingredient content of *B. striata* and *B. ochracea*. Comparison of morphological measurements (**a**), pseudobulb weight (**b**) and the main active ingredient contents (**c**) in different growth stages and different species of *B. striata* and *B. ochracea*. Values are the mean ± SD (standard deviation), and different lowercase letters indicate significant differences with the parameter of *P* < 0.05

The morphological phenotypes of the aboveground parts of 33 populations (including 28 *B. striata* populations and 5 *B. ochracea* populations) were measured for three years (Fig. 1; Table S2). Statistical analysis showed that there were abundant phenotypic variations and significant differences among different populations, especially the plant height, leaf blade length, stem diameter and inflorescence height with high F values and extremely low *P* values (Table 1). The plant height, leaf blade length, inflorescence height and length of *B. ochracea* were greater than those of *B. striata*. In addition to leaf blade length in *B. striata*, the plant height, leaf blade length and width of *B. striata* and *B. ochracea* increased with increasing planting years, while the fruit number decreased in *B. ochracea* and *B. striata* (Fig. 2a).

**Table 1:** Summary of the main statistical parameters in the one-way ANOVA of phenotypic traits and the main active ingredient contents

		**Min**	**Max**	**Mean**	**CV (%)**	**F**	***P***	***n***
Plant height (cm)	2018	5	57	25.79	38.61	19.62	1.08E-72	621
	2019	10	80	29.78	28.26	27.26	2.89E-85	482
	2020	13	68	32.32	33.33	53.19	1.14E-150	617
Leaf blade length (cm)	2018	7	55	29.79	24.99	15.99	1.61E-60	621
	2019	10	54	30.02	24.16	31.67	7.39E-95	482
	2020	10	64	30.21	27.71	33.28	3.67E-110	617
Leaf blade width (cm)	2018	1.0	12.5	4.51	40.00	22.83	2.15E-82	617
	2019	2.0	9.5	4.85	28.99	26.51	1.49E-83	482
	2020	2.0	11.5	5.16	27.66	23.13	2.84E-83	617
Leaf number	2018	1	6	3.69	19.79	5.87	3.49E-20	621
	2019	2	8	4.30	23.41	11.51	6.83E-41	482
	2020	2	43	4.01	43.04	1.53	3.26E-02	617
Stem diameter (mm)	2018	0.33	9.86	5.31	33.84	15.69	1.75E-59	621
	2019	2.13	11.36	5.44	26.19	32.96	1.72E-97	482
	2020	2.08	9.85	5.75	23.05	21.41	4.17E-78	617
Inflorescence height (cm)	2018	15	71	39.83	29.67	13.65	1.35E-38	326
	2019	10	89	32.11	35.96	37.76	2.58E-105	470
	2020	19	83	40.35	27.98	37.33	2.05E-119	616
Inflorescence length (cm)	2018	11	63	32.08	33.57	13.69	9.76E-39	327
	2019	10	69	30.27	30.51	31.96	2.81E-94	470
	2020	12	58	32.51	28.55	33.26	4.91E-110	616
Flower number	2018	3	17	9.00	33.27	5.19	6.49E-14	325
	2019	1	14	5.85	46.54	26.98	1.49E-83	469
	2020	3	17	8.83	25.70	20.16	5.61E-74	611
Fruit number	2018	1	9	2.60	51.26	1.47	6.85E-02	244
	2019	1	5	1.47	47.06	3.70	5.89E-09	280
	2020	3	3	1.49	50.59	7.46	1.14E-20	263
Weight of pseudobulb (g)	2017	1.4	111.0	34.27	69.55	38.75	1.23E-93	370
	2018	3.2	281.1	81.92	66.25	12.05	1.37E-05	278
	2019	6.7	725.0	138.97	72.34	13.21	2.55E-22	286
	2020	5.8	597.0	185.32	71.06	14.28	5.29E-89	270
Polysaccharide content (%)	2017	18.36	59.38	45.03	18.92	604.53	0.00E + 00	99
	2018	15.36	60.00	45.66	18.68	740.31	0.00E + 00	99
	2019	12.37	59.60	46.94	21.03	880.41	1.37E-34	99
	2020	8.34	55.31	42.84	27.16	2343.25	3.01E-02	99
Total phenol content (%)	2017	1.57	4.84	3.73	21.00	481.12	3.84E-05	99
	2018	1.36	5.07	3.63	22.61	427.51	1.42E-33	99
	2019	0.84	4.97	3.77	23.47	334.64	0.00E + 00	99
	2020	1.21	5.01	3.42	29.48	312.33	0.00E + 00	99
Militerane content (%)	2017	0.45	3.99	1.44	46.62	602.31	6.51E-38	99
	2018	0.51	3.68	1.43	45.93	670.60	2.38E-01	99
	2019	0.55	3.11	1.51	40.05	469.44	1.67E-02	99
	2020	0.69	4.29	1.60	44.19	284.97	7.44E-38	99

*Min and Max* the minimum value and maximum value in the sample; Mean, the average value of the sample, *CV (%)* coefficient of variation; F, ratio of MSA/MSE, *P* P value; n, sample size

There were significant differences in pseudobulb weight among different species and different populations, and the coefficient of variation (CV) of pseudobulb weight was higher than the CVs of other phenotypic traits (Fig. 2, Table 1). In detail, the weight of the one-year-old pseudobulb of different populations varied from 5.02 to 86.08 (g), and the CV (%) was 69.55. Equal group classification method reveals that population categorized in class 5 represents the maximum level of pseudobulbs weight, and GXHZ, HBXG and HBLT populations were categorized in class 5, while the last five populations were HBXS, HBSY, HBWF (*B. ochracea*), SXXX and GXLY, which were categorized in class 1. For the two-year-old pseudobulb, the weight in different populations varied from 12.58 to 176.35 (g), and the CV (%) was 66.25. GXHZ, HBXG, HBLT, AHBZ and GZZY were ranked as the top five populations, which were categorized in class 5, while the last five populations were SCLS, HBSY, HBWF (*B. ochracea*), SXXX and GXLY. For the three-year-old pseudobulb, the weight in different populations varied from 23.13 to 329.42 (g), and the CV (%) was 72.34. GXHZ, AHBZ, HBXG and HBLT were categorized in class 5, while the last five populations were SYZS, HBSY, HBWF (*B. ochracea*), SXXX, and GXLY. For the four-year-old pseudobulb, the weight in the different populations varied from 31.63 to 442.77 (g), and the CV (%) was 71.06. HBLT, AHBZ, HBWH and HBSN were categorized in class 5, while the HBWF (*B. ochracea*), HBSY, SXXX, HNCL and GXLY populations showed lower weights. For pseudobulb weight, HBLT, AHBZ, HBXG, GXHZ and HBSN populations can be prioritized (Table S3). In general, except in GXHZ, HNCL and SCWY, the weight of the pseudobulbs increased with the growing period, while the average annual growth rates of the pseudobulb were 2.42, 1.69 and 1.31, respectively, showing a decreasing trend. There was a significant difference between the two species, and the average one-, two-, three- and four-year-old pseudobulb weights of *B. ochracea* were 19.66, 63.26, 102.07 and 140.95 (g), which were lower than the 37.44, 85.72, 144.79 and 193.49 (g) of *B. striata*, respectively (Table 2, Fig. 2a).

**Table 2:**
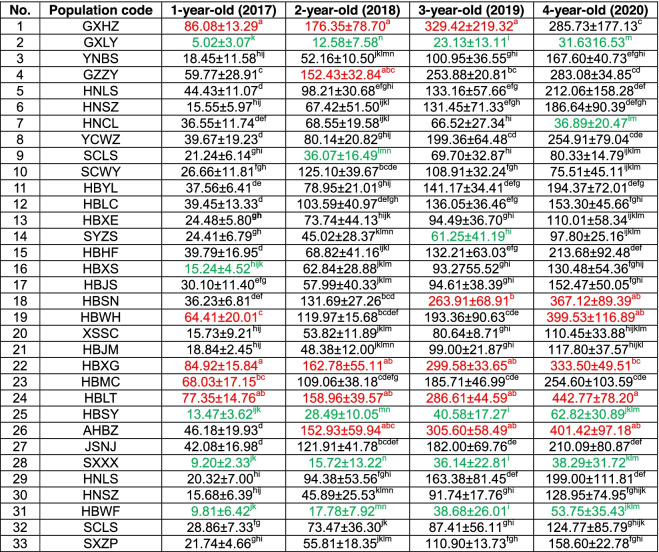
The fresh weight of pseudobulbs of 28 populations of *B. striata* and 5 populations of *B. ochracea*

No. 1–28 are *B. striata*, No. 29–33 are *B. ochracea*. Red represents the top five populations of pseudobulb weight in different years, while green represents the last five populations. Values represent the mean ± SD of ten individuals, and different lowercase letters with superscripts indicate significant differences between populations with the parameter of *P* < 0.05

## Determination of active ingredients content

The polysaccharide, total phenol and militarine contents of pseudobulbs were determined among different species and populations. The results showed that significant differences were observed among different populations, with a minimum F value of 284.97 and *P* values much lower than 0.05 (Table 1). Overall, there were no differences in the content of the main active ingredients among different species and different planting years (Fig. 2c).

Most populations had an excellent phenotype in terms of polysaccharide content, which varied from 8.52 to 59.77 (%). There are 8, 12, 15 and 13 populations accounted for more than 50% of the one-, two-, three- and four-year-old pseudobulbs, and 17, 12, 13 and 9 populations ranged from 40 to 50%, respectively (Table 3). Equal group classification shows that most populations were categorized in class 4 and class 5 (Table S5).The average polysaccharide contents of one-, two-, three- and four-year-old pseudobulbs of *B. ochracea* were 45.18, 47.57, 44.70 and 40.45 (%), and the average polysaccharide contents of one-, two-, three- and four-year-old pseudobulbs of *B. striata* were 45.00, 45.32, 47.34 and 42.92 (%), which means the content of polysaccharide in pseudobulb of *B. striata* was three-year-old > two-year-old > one-year-old > four-year-old, while which of *B. ochracea* was two-year-old > one-year-old > three-year-old > four-year-old (Fig. 2c). In general, the top five populations in class 5 with the highest polysaccharide content in the pseudobulbs of the average of four years were HBYL, SXZP (*B. ochracea*), AHBZ, HBHF and HBWF (*B. ochracea*), while HNSZ (*B. ochracea*) and YNBS were categorized in class 2 and class 1, respectively (Table S5).

**Table 3:**
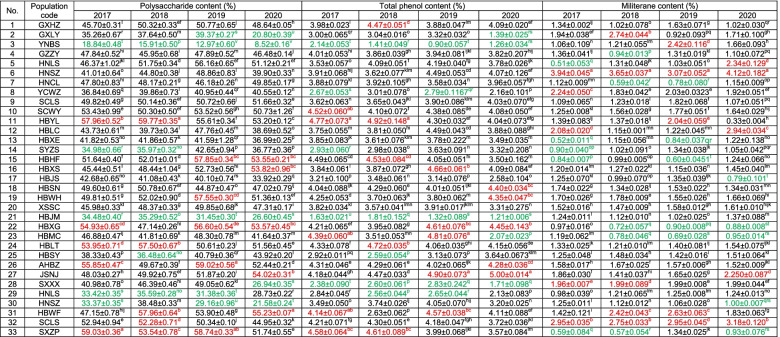
Active ingredient contents of 28 populations of *B. striata* and 5 populations of *B. ochracea*

No. 1–28 are *B. striata*, No. 29–33 are *B. ochracea*. Red represents the top five populations in terms of active ingredient contents in different years, while green represents the last five populations. Values represent the mean ± SD of three biological replicates, and different lowercases letters with superscript indicate significant differences between populations with the parameter of *P* < 0.05

Obvious variation was observed in the content of total phenol, which varied from 0.90 to 5.00 (%). There were 14, 11, 16 and 13 populations that were higher than 4% of the one-, two-, three- and four-year-old pseudobulbs, and 12, 15, 12 and 12 populations ranged from 3 to 4%, respectively (Table 3). The average total phenol content of one-, two-, three- and four-year-old pseudobulbs of *B. ochracea* were 3.96, 3.57, 3.89 and 3.35 (%), which were lower than the 3.98, 4.14, 4.33 and 3.43 of *B. striata*, respectively. Therefore, the order of the contents of total phenol in the pseudobulb of *B. striata* was three-year-old > two-year-old > one-year-old > four-year-old, while that in *B. ochracea* was one-year-old > three-year-old > two-year-old > four-year-old (Fig. 2c). In general, the top five populations in class 5 with the highest average total phenol contents in pseudobulbs of four years were JSNJ, HBYL, HBXG, SCWY and HBLT, while YNBS and HBJM populations were categorized in class 1 (Table S6).

The average milliarine contents of the one-, two-, three- and four-year-old pseudobulbs of *B. ochracea* were 1.43, 1.62, 1.85 and 1.64 (%), which were generally higher than those of *B. striata* (1.44, 1.39, 1.45 and 1.60 (%), respectively). That is, the order of the militarine contents of the pseudobulbs of *B. striata* was four-year-old > three-year-old > one-year-old > two-year-old, while that in *B. ochracea* was three-year-old > four-year-old > two-year-old > one-year-old (Fig. 2c). Militarine contents varied from 0.51 to 4.12 (%), 4, 4, 6 and 5 populations accounted for more than 2% of the one-, two-, three- and four-year-old pseudobulbs, and 7, 5, 9 and 12 populations accounted for 1.5% to 2%, respectively (Table 3). Overall, most populations have low militerane content, there are 19 populations were categorized in class 1. The HNSZ, SCLS (*B. ochracea*), HBWF (*B. ochracea*) and YCWZ, which were categorized in class 3 to 5 can be prioritized, while the HBHF, HNCL, HBMC, HBXG and SXZP (*B. ochracea*) populations in class 1 were the five populations with the lowest contents (Table S7).

GRA of the pseudobulb weight, pseudobulb growth ratio and main active ingredient content showed that there are 2 populations greater than 0.6 and 15 populations greater than 0.5. Meanwhile, HNSZ, AHBZ, HBLT, HBSN and JSNJ in class 5 were ranked as the top five, indicating that these populations have good characteristics in comprehensive yield and quality, while the last five populations of class 1 were SXXX, SYZS, GXLY, HBJM and YNBS (Table 4, Table S8). Meanwhile, HCA showed that HBWH, HBSN, AHBZ and HBLT belonged to group I, and GZZY, HBXG and GXHZ belonged to group II when the parameters were chosen to divide the population into 5 categories (Fig. S1).

**Table 4:** Correlation degree and rank of pseudobulb weight and main active ingredient contents based on gray relational analysis

Population code	Correlation degree		Relative correlation degree	Rank
	**relative to optimal seq**	**relative to least seq**		
HNSZ	0.721	0.432	0.625	1
AHBZ	0.683	0.434	0.611	2
HBLT	0.676	0.471	0.589	3
HBSN	0.631	0.452	0.583	4
JSNJ	0.628	0.465	0.574	5
GXHZ	0.635	0.501	0.559	6
HBWH	0.611	0.483	0.558	7
SCLS (*B. ochracea*)	0.607	0.485	0.556	8
HBXG	0.649	0.525	0.553	9
HBYL	0.639	0.532	0.545	10
SCWY	0.608	0.508	0.545	11
GZZY	0.566	0.505	0.528	12
HBXS	0.577	0.522	0.525	13
SXZP (*B. ochracea*)	0.595	0.558	0.516	14
HNLS	0.552	0.538	0.506	15
HBLC	0.528	0.518	0.505	16
HBHF	0.571	0.568	0.501	17
HBWF (*B. ochracea*)	0.574	0.575	0.500	18
XSSC	0.525	0.533	0.496	19
YCWZ	0.488	0.531	0.479	20
HBMC	0.531	0.590	0.474	21
HNLS (*B. ochracea*)	0.501	0.571	0.467	22
SCLS	0.515	0.597	0.463	23
HBXE	0.476	0.593	0.445	24
HNSZ (*B. ochracea*)	0.467	0.599	0.438	25
HNCL	0.494	0.652	0.431	26
HBJS	0.451	0.612	0.424	27
HBSY	0.445	0.633	0.413	28
SXXX	0.446	0.655	0.405	29
SYZS	0.438	0.646	0.404	30
GXLY	0.440	0.665	0.398	31
HBJM	0.406	0.694	0.369	32
YNBS	0.409	0.757	0.351	33

The populations of unmarked species are *B. striata*

### Correlation analysis of phenotypic traits, pseudobulb weight and active ingredient content

**Fig. 3:**
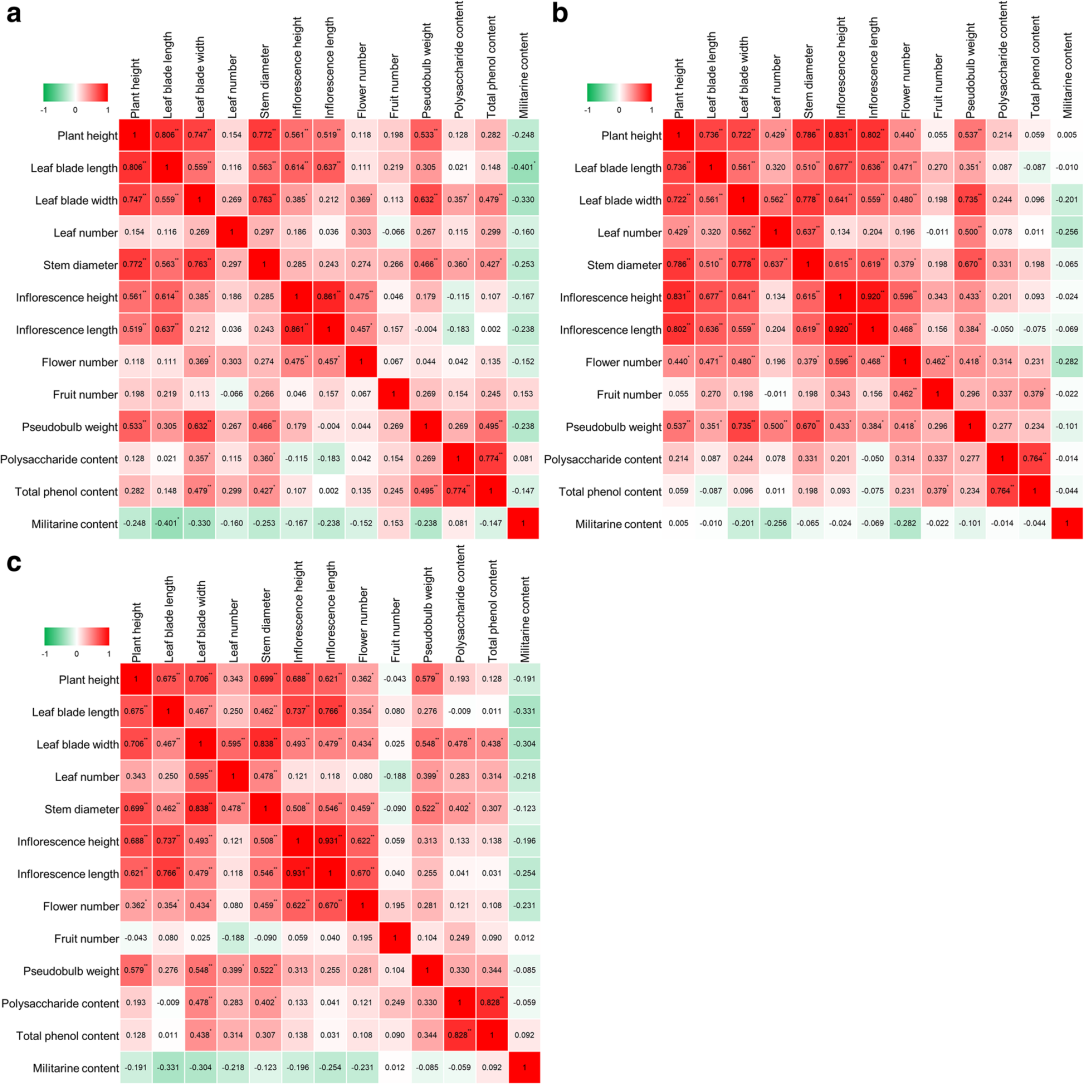
Correlation analysis of phenotypic traits, pseudobulb weight and active ingredient contents. Correlation analysis of phenotypic traits, pseudobulb weight and main active ingredient contents in 2018 (**a**), 2019 (**b**) and 2020 (**c**) was carried out. Values are Pearson correlation coefficients, which are used to construct the heat map. * or ** represent a significant difference with the parameters of *P* < 0.05 and *P* < 0.01, respectively

To investigate which phenotypic traits may determine the yield of pseudobulb and active ingredient content, and whether there is a correlation between the yield of pseudobulb and active ingredient content, Pearson correlation analysis was performed for two-, three- and four-year-old plants among 13 indexes, including 9 morphological indexes of aboveground parts, pseudobulb weight indexes and 3 active ingredient indexes. The results showed that except for the negative correlation between the militarine content and other indexes, the other 12 indexes were positively correlated (Fig. 3).

The correlation coefficient of plants in 2018 ranged from -0.401 to 0.861. The correlation coefficients between plant height and leaf blade length, and between inflorescence height and inflorescence length were greater than 0.8 (0.806 and 0.861, respectively). There were 4 other correlation coefficients greater than 0.7, 3 correlation coefficients greater than 0.6, 5 correlation coefficients greater than 0.5, and 7 correlation coefficients greater than 0.4, and all of them showed extremely significant differences with *P* values of less than 0.01. Overall, there were significant correlations between phenotypic traits except leaf number, flower number and fruit number. There were significant correlations between pseudobulb weight and plant height, leaf blade width, stem diameter, and total phenol content. There were significant correlations between polysaccharide content and leaf blade width, stem diameter, and total phenol content. There were significant correlations between the total phenol content and leaf blade width, stem diameter, pseudobulb weight, and polysaccharide content. There was a significant negative correlation between militarine content and leaf blade length (Fig. 3a).

The correlation coefficient of plants in 2019 ranged from -0.282 to 0.920. The correlation coefficient of inflorescence height and inflorescence length was 0.920. The correlation coefficients of plant height with inflorescence height and inflorescence length were greater than 0.8. There were 6 other correlation coefficients greater than 0.7, 7 correlation coefficients greater than 0.6, and 7 correlation coefficients greater than 0.5, and all of them had extremely significant differences with a *P* value less than 0.01. Overall, there were significant correlations between phenotypic traits except leaf number, and fruit number. There were significant correlations between pseudobulb weight and all phenotypic traits except for fruit number; however, the three indexes with the strongest correlations were the same as those in 2018, which were plant height, leaf width and stem diameter. The correlation between polysaccharide content and phenotypic traits was lower than that in 2018, and the same trends were observed for total phenol and militerine. However, there was still a significant correlation between the polysaccharide and total phenol content (Fig. 3b).

The correlation coefficient of plants in 2020 ranged from -0.331 to 0.931, and the highest value was obtained for inflorescence height and inflorescence length. There were 2 other correlation coefficients greater than 0.8, 3 correlation coefficients greater than 0.7, 6 correlation coefficients greater than 0.6, and 6 correlation coefficients greater than 0.5, and all of them had extremely significant differences with a *P* value less than 0.01. Overall, there were significant correlations between phenotypic traits except leaf number and fruit number. There were significant correlations between pseudobulb weight and plant height, leaf blade width, leaf number, and stem diameter. There were significant correlations between polysaccharide content and leaf blade width, stem diameter, total phenol content. There were significant correlations between the total phenol content and leaf blade width, and polysaccharide content. There was also a nearly negative correlation between the militarine content and other indexes (Fig. 3c).

## Discussion

Stable yield and quality are the most important value indicators of medicinal materials, and good germplasm resources are the basis for ensuring the quality of medicinal materials. In our research, we continuously evaluated the main value species, including *B. striata* and *B. ochracea*, for 4 years. Based on our yearly results, we screened excellent populations with higher yields in terms of four-year-old pseudobulb weight (Table 2, Table S3-4) and higher contents of polysaccharide, total phenol and militarine compounds, respectively (Table 3, Table S5-7), indicating that these populations were good germplasm resources for the single-component utilization of *Bletilla*. Furthermore, HNSZ, AHBZ, HBLT, HBSN and JSNJ populations in class 5 showed good characteristics in terms of pseudobulb weight, the pseudobulb growth ratio and the main active ingredients contents according to GRA, indicating that these populations showed higher quality with good values of all the indicators (Table 4, Table S8). Due to the difference in calculation method, PCA showed that HBWH, HBSN, AHBZ and HBLT in group I, and GZZY, HBXG and GXHZ in group II were high placing. Combining the two analyses, AHBZ, HBLT and HBSN populations can be prioritized.

*Bletilla* is not strictly geoauthentic medicinal plant. The different geographical populations from different provinces may have good quality and are excellent germplasm resources. Chen et al. also found that *Bletilla* from different provinces of China have similar HPLC fingerprints, and were classified into one group by cluster analysis [28]. In addition, even in the same region, there may be a large difference between different geographical populations. Western Hubei Province is rich in forest resources, which provide good ecological conditions for the breeding and reproduction of medicinal plants. We collected the HBXS, HBSN, XSSC and HBYL populations near the Shennongjia Forestry District and the HBLC, HBXE, HBHF, HBJS and HBWF (*B. ochracea*) populations near the Enshi Tujia and Miao Autonomous Prefecture. We found that there were differences in the weight of pseudobulbs and the contents of three active ingredients among different populations, whether near Shennongjia Forest District or Enshi Tujia and Miao Autonomous Prefecture, and there were abundant variations among populations. For example, the weights of the pseudobulbs of the HBXS and XSSC populations were significantly lower than those of HBSN, and the polysaccharide and total phenol contents in HBXS were higher than those in HBSN. The content of militerine was highest in HBLC, while that in HBXE and HBHF was very low, and the HBHF population had high contents of polysaccharide and total phenol. The determination of total polyphenol in *B. striata* from 16 areas of northwestern of Hubei Province showed that the content of total polyphenol varied in different areas. The analysis of the polysaccharide content of 18 samples of *B. formosana* from 8 populations in Baoshan City of Yunnan Province showed that there were significant differences among different samples. The polysaccharide contents of *B. striata* from different areas of Guizhou and Anhui Province were also significantly different [18]. All these studies showed that it is necessary to understand the resource distribution, habitat and situation of *Bletilla* and then to evaluate germplasm resources, which can provide a theoretical basis for the protection and utilization of *Bletilla*.

Traditionally, although other species of *Bletilla* are also used as local medicinal plants, *B. striata* is the only species officially approved for use [3]. Considering the high medicinal value, high demand and gradually diminishing wild resources of *B. striata*, to protect traditional resources and develop new medicinal plant resources, it is necessary to conduct in-depth research on *B. striata* and its related species, to excavate alternative resources of *B. striata* and reduce the waste of effective ingredients and resources. However, the distribution of *B. sinensis* is narrow and limited to the south of Yunnan Province in China. The pseudobulb size of *B. formosana* is significantly smaller than the pseudobulb size of *B. ochracea* and *B. striata*, and the polysaccharide content of *B. formosana* was slightly lower than the polysaccharide content of *B. ochracea* and *B. striata* [29]. The pseudobulbs of *B. ochracea* are larger, and its flowering period is later than the flowering period of *B. striata*. Currently, *Bletilla* is widely planted as a bonsai plant, so in addition to ornamental plants, can underground pseudobulbs of *B. ochracea* also be harvested as medicinal plants? We chose 5 populations of *B. ochracea* and 28 populations of *B. striata* to demonstrate the phenotypic quality of *B. striata* with higher average pseudobulbs weights, while there was no difference between the two species in terms of polysaccharide, total phenol and militarine contents. These results suggested that *B. ochracea* could be accepted as an alternative resource to *B. striata.*

Efficient screening of excellent genetic resources is important for new germplasm creation and breeding, and efficiently discovering the phenotypic indicators significantly related to yield and quality is especially critical for perennial herbs [30]. Usually, *B. striata* exhibits the best comprehensive traits and is harvested in the third year after cultivation [31, 32], which requires at least 3 years to estimate the genetic material and preliminarily screen out a quality germplasm. In this paper, Pearson correlation analysis of two-, three- and four-year-old plants showed that there were significant correlations between pseudobulb weight and other phenotypic traits, especially plant height, leaf blade width and stem diameter, which implies that plants with excellent growth can produce better pseudobulb yields (Fig. 3). Interestingly, there was a strong significant correlation between the polysaccharide and total phenol contents, and polysaccharide and total phenol showed a similar trend to pseudobulb yield, which was significantly related to leaf blade width and stem diameter. These results may provide important and more direct indicators for quickly screening and breeding potential good germplasms. The uncommon positive correlation between yield and the main quality factors may provide hopeful prospects for excellent germplasm breeding of *Bletilla* herbs; of course, this positive relation and the genetic foundation of this relation need further demonstration and in-depth investigation [26]. In addition, the militarine content was almost negatively correlated with other indexes, which indicated that the accumulation of militarine may occur more often under the condition of limited growth. We isolated one-, two-, three- and four-year-old pseudobulbs of four-year-old plants from four populations. The militarine content in pseudobulbs shows an increasing trend year by year, implying that sufficient growth years are required to accumulate more militarine content, and further research is needed to reveal the synthesis and metabolic mechanisms of militarine in *Bletilla* plants (unpublished). Next, integrating phenomics, genomics and metabonomics approaches for high-throughput mining of the key phenotypes and revealing the genetic mechanism is our aim in recent years.

The growth capacity of yield characteristics can also be selected to evaluate different genetic resources. Populations of HBSN, HBWH, and AHBZ showed excellent yield phenotypes with high yield and growth capacity, and the pseudobulb weight of the HBSN population could even reach 7.29 and 10.13 times in the third and fourth years after cultivation, respectively. Other populations such as HNSZ, SXZP (*B. ochracea*), HBXS, and HBJM, also showed a good growth capacity. Our results indicated that the mean annual growth ratios of pseudobulbs in 3 years were 2.42, 1.69 and 1.31, which gradually decreased with the cultivation years. Even in some populations, such as GXHZ, HNCL and SCWY, the pseudobulb weight in the fourth or even third year began to decrease. Yearly content variation showed that the average contents of both polysaccharides and total phenol in the one-, two-, three- and four-year-old pseudobulbs of *Bletilla* varied in the following order: three years old > two years old > one year old > four years old, which confirmed the conclusion regarding the best harvest period of *Bletilla* [31, 32]. However, the average militarine content of one-, two-, three- and four-year-old pseudobulbs of *Bletilla* varied in the following order: four-year-old > three-year-old > one-year-old > two-year-old, which again indicated differences in polysaccharide and total phenol, and the synthesis and metabolic mechanisms of militarine in *Bletilla* plants need to be revealed.

## Conclusion

There were abundant phenotypic variations and significant differences among different populations in the morphological phenotypes, pseudobulb weight and main active ingredient contents. *Bletilla* is not strictly geoauthentic medicinal plants. Excellent germplasm resources may be distributed in distant geographical locations. There may be a large difference between different geographical populations even in the same region. *B. ochracea* could be accepted as an alternative resource to *B. striata*. AHBZ, HBLT and HBSN populations showed good prospects for industrial development, presenting higher quality in terms of yield and main active ingredient content. Pseudobulb yield, polysaccharide and total phenol content are positively correlated with phenotypic traits. Militarine content is negatively correlated with almost all indexes. Plant height, leaf width and stem diameter may be important indicators of potential excellent germplasms. The best harvest period of *Bletilla* is the third year after cultivation. These results provide important information required for the efficient screening and utilization of *Bletilla* germplasm resources.

## Methods

### Experimental material

Thirty-three natural populations of *Bletilla* (including 28 *B. striata* populations and 5 *B. ochracea* populations) were randomly collected from its main natural range across ten provinces in China (Table S1; Fig. 1). In 2017, 30 individuals of new pseudobulb of the year with similar size were collected from each population without affecting the continuous growth of wild resources and transported to a germplasm resources nursery in the Wuhan Botanical Garden (WBG) of the Chinese Academy of Sciences (CAS) for common-garden cultivation with the same soil formula of 20% sand, 30% humus and 50% garden soil.

WBG is an institution for species conservation and scientific research. The ex-situ conservation of plant resources is the main job of WBG. Introducing species from other area only requires a work certification issued by WBG and does not require government or local administrative permission. The representative specimens of plants from each population were identified by XiaoDong Li and deposited in the herbarium (HIB, 0,164,057–01,640,144; 0,184,514; 0,168,975–0,168,979; 0,202,319; 0,202,331–0,202,332; 0,144,060; 0,149,075–0,149,076; 0,150,992–0,150,993; 0,164,047–0,164,048, *Bletilla striata* (Thunb. ex A. Murray) Rchb. f.) of WBG. The collected samples are only for scientific research and comply with the guidelines of WBG.

### Phenotypic measurements

The weight of the pseudobulb was measured and marked as one-year-old in winter 2017. The morphology of the aboveground parts and the weight of the pseudobulbs of 10 individuals were measured every year for the next three years (2018–2020).

The recorded morphological parameters of the aboveground parts included plant height (cm), leaf blade length (cm), leaf blade width (cm), total leaf number, stem diameter (mm), inflorescence height (cm), inflorescence length (cm), total flower number and total fruit number. Leaf phenotypes and stem diameter were measured in August or September. Plant height was the vertical distance between the top of the highest leaf and the ground. Leaf length and leaf width were measured using the largest leaf. The phenotypes of the inflorescences were measured in April or May. Inflorescence height was the vertical distance from the base to the tip of the inflorescence stem.

In early November 2018, the withered aboveground parts of 10 individuals were removed, and the pseudobulbs (two years old) were excavated and cleaned to measure the weight. The corresponding fraction of pseudobulbs was removed for the subsequent determination of polysaccharide, total phenol and militarine content. The same method was used to measure the weight of pseudobulbs in 2019 (three years old) and in 2020 (four years old) using the other 10 individuals.

### Polysaccharide content analysis

After removing the fibrous roots, the sampled pseudobulbs were sliced, steamed in a water bath, and dried in the shade according to the Chinese Pharmacopeia. Dried pseudobulb slices were crushed for polysaccharide content, total phenol content and militarine content analyses. All the samples were collected in early November 2017, 2018, 2019 and 2020.

The phenol–sulphuric acid method was used to analyze polysaccharide content as described previously with minor modifications [23]. Approximately 2 g of dried pseudobulb powder was treated with 50 mL H_2_O for 120 min at 100 °C twice using the conventional heating reflux extraction method. The 100 mL extract was concentrated to 10 mL by rotary evaporation, 25 mL of 95% ethanol solution was added to half of the extract solution (5 mL) for precipitation, and they had to rest overnight at room temperature. After centrifugation (3000 rpm/min) for 15 min, the precipitate was dissolved in 25 mL H_2_O to obtain the crude polysaccharide solution and quantified by the phenol–sulfuric acid method. 10 mg of standard glucose was dissolved in 100 mL H_2_O at a concentration of 100 µg/mL and diluted to six different concentrations of 0, 20, 40, 60, 80 and 100 µg/mL. 1 mL glucose solution, 1 mL 5% phenol and 6 mL concentrated sulfuric acid were added into a plug test tube in turn. After shaking and mixing, the samples were left at 25 °C for 40 min, and the absorbance was determined at 484 nm (A_484_). The regression equation was A_484_ = 0.0088 C (µg/mL) + 0.0122, *r* = 0.9998 (*n* = 6). Then, 1 mL polysaccharide solution was measured by the same method as glucose solution. The absorbance was compared with the regression equation, multiplied by the dilution multiple (50) and divided by the weight of dried pseudobulb powder. The results were expressed as polysaccharide content (%).

### Total phenol content analysis

The sampling procedure was the same as above. Total phenol content was determined by Folin-Ciocalteu’s phenol reagent [33]. Gallic acid was used as the standard to draw the regression equation, and 100 mg gallic acid was dissolved in 100 mL 60% ethanol at a concentration of 1000 µg/mL and diluted to five different concentrations of 10, 20, 30, 40 and 50 µg/mL. Then, 1 mL of gallic acid solution and 5 mL 10% (v/v) Folin-Ciocalteu’s phenol reagent were added into a test tube to react for 10 min, and 5 mL 2% (m/v) Na_2_CO_3_ was added. After shaking, the sample was allowed to rest at 25 °C for 1 h, and the absorbance of each sample was determined at 760 nm (A_760_). The regression equation was A_760_ = 0.0132 C (µg/mL) − 0.0004, *r* = 0.9999 (*n* = 6). Approximately 2 g dried pseudobulb powder was treated with 80 mL 60% ethanol for 120 min at 90 °C by using the conventional heating reflux extraction method. Cooling down to RT, the solution was made up to 80 ml with 60% ethanol and then filtered using filter paper. The residue was extracted with 60% ethanol again, and the filtrates were mixed as the test solution, which was measured by the same method as the standard sample, to obtain the A_760_, compared with the regression equation, multiplied by the dilution multiple (160) and divided by the weight of dried pseudobulb powder. The results were expressed as total phenol content (%).

### Militarine content analysis

The sampling procedure was the same as above. HPLC was used to measure the militarine content according to the Chinese Pharmacopeia 2020 edition. 11.4 g millitarine was dissolved in 6 ml 52.9% ethanol to obtain a 1.9 mg/mL standard solution, which was diluted to six different concentrations of 1900, 950, 475, 237.5, 118.75 and 59.375 µg/mL. The standard solutions were analyzed by reversed-phase HPLC using a mobile phase of acetonitrile and water with 0.1% phosphoric acid (elution ratio: 22:78, v/v; wavelength: 223 nm; flow rate: 1 mL/min; injection volume: 10 µL) and the column oven temperature was set at 30 °C. The regression equation of the standard curve was peak area = 22,287,199 C (µg/mL) + 137,858, *r* = 1 (*n* = 6). Approximately 0.2 g dried pseudobulb powder was extracted with 25 ml 52.9% ethanol at 30 °C for 30 min by an ultrasound system (power = 300 W, frequency = 37 Hz). The solution was brought up to 25 ml with 52.9% ethanol and then filtered through filter paper. The filtrate was analyzed by the same method as the standard sample, compared with the regression equation, multiplied by the dilution multiple (25,000) and divided by the weight of the dried pseudobulb powder. Ultimately, the results were expressed as militarine content (%).

### Prioritization and elite identification

The value of each parameter, including pseudobulbs weight, pseudobulb growth ratio, polysaccharide content, total phenols content, militerane content and GRA (Tables 2, 3, and 4), in each population was categorized into five equal classes, and population in class 5 with higher values were considered as a prioritized group of the population [34]. At the same time, the sum of the one-, two-, three- and four-year-old pseudobulb weight, and the average of polysaccharide, total phenols and militerane content in one-, two-, three- and four-year-old pseudobulb were used for prioritization and elite identification.

### Gray relation analysis

The means of the values in Tables 2 and 3 were used as the raw data for GRA. For pseudobulb weight, in addition to considering the weight of the pseudobulb measured directly in every year, pseudobulb growth ratios of one, two and three years were also used as indexes. A total of 19 indexes constituted the evaluation matrix, {*X*_*ik*_} (*i* = 1, 2, 3, … m; k = 1, 2, 3, … n, m = 33, *n* = 19) and calculated the average value (‾*X*_*K*_) of each index. The original data were normalized by the formula *Y*_*ik*_ = *X*_*ik*_/‾*X*_*K*_. The maximum and minimum values of each index were selected as the optimal {*Y*_*sk*_} and the least {*Y*_*tk*_} reference sequences respectively. The correlation coefficients relative to the optimal and the least reference sequences were calculated with the formulas (∆min + ρ∆max)/(*Y*_*sk*_—*Y*_*ik*_ + ρ∆max) and (∆min + ρ∆max)/(*Y*_*ik*_—*Y*_*tk*_ + ρ∆max), respectively, where ρ is the discrimination coefficient, and its value is generally approximately 0.5, ∆min = min | *Y*_*sk*_ – *Y*_*ik*_ |, ∆max = max | *Y*_*sk*_ – *Y*_*ik*_ |. The uniform average of the correlation coefficient in each index was used to calculate the correlation degree relative to the optimal and the least reference sequences, which is expressed as *r*_*i(s)*_ and *r*_*i(t)*_, respectively. The relative correlation degree (*r*_*i*_) of the evaluation matrix {*X*_*ik*_} relative to the optimal and least reference sequences was defined as *r*_*i*_ = *r*_*i(s)*_ / (*r*_*i(s)*_ + *r*_*i(t)*_), which wes used to rank (Table 4).

### Hierarchical clustering analysis (HCA)

The data used for GRA (pseudobulb weight, pseudobulb growth ratio, polysaccharide content, total phenol content and militarine content) was also used for HCA by IBM SPSS Statistics 25 software.

### Correlation analysis

The means of the values in Table S2, Tables 2 and 3 were used to calculate the Pearson correlation coefficient with IBM SPSS Statistics 25 software, which was used to construct a heat map in Microsoft Office Excel 2019. * or ** represent significant differences with the parameters of *P* < 0.05 and *P* < 0.01, respectively.

### Data analysis

Two-tailed ANOVA was performed to test significant differences in measured variables among different populations and different growth years. ANOVA and Pearson correlation analysis were carried out with IBM SPSS Statistics 25.

## Supplementary Information

**Additional file 1: Table S1.** Detailed distribution information of the Bletilla plants collected from China.

**Additional file 2: Table S2.** Variation in phenotypic characteristics of thirty-three geographical populations of *Bletilla*. N/A indicates the population did not grow inflorescence or fruit in that year.

**Additional file 3: Table S3 **Ranking and identification of promising population based on pseudobulbs weight. **Table S4.** Ranking and identification of promising population based on pseudobulb growth ratio. **Table S5.** Ranking and identification of promising population based on polysaccharide content. **Table S6.** Ranking and identification of promising population based on total phenols content. **Table S7.** Ranking and identification of promising population based on militerane content. **Table S8.** Ranking and identification of promising population based on GRA. **Fig. S1.** Hierarchical clustering analysis of pseudobulb weight, pseudobulb growth ratio and main active ingredient content.

## Data Availability

All data generated or analysed during this study are included in this published article [and its supplementary information files].

## Abbreviation

GRA: Gray relation analysis
