# Supplementary material for: Phenotype correlation analysis and excellent germplasm screening of herb Bletilla Rchb. f. based on comprehensive evaluation from thirty-three geographic populations
Source: BMC Plant Biol. 2022 Mar 29;22:154. doi: 10.1186/s12870-022-03540-w (PMC8966332; doi:10.1186/s12870-022-03540-w)

**Table S3 Ranking and identification of promising population based on pseudobulbs weight**

| **Class** | **Range** | **Mean** | **Populations** | **Number** | |
| --- | --- | --- | --- | --- | --- |
| One-year-old pseudobulbs weight (2017) | | | | |  |
| class 1 | 5.02 - 21.23 | 14.31 | GXLY, SXXX, HBWF (*B. ochracea*), HBSY, HBXS, HNSZ, HNSZ (*B. ochracea*), XSSC, YNBS, HBJM, HNLS (*B. ochracea*) | 11 | |
| class 2 | 21.23 - 37.45 | 27.81 | SCLS, SXZP (*B. ochracea*), SYZS, HBXE, SCWY, SCLS (*B. ochracea*), HBJS, HBSN, HNCL | 9 | |
| class 3 | 37.45 - 53.66 | 41.31 | HBYL, HBLC, YCWZ, HBHF, JSNJ, HNLS, AHBZ | 7 | |
| class 4 | 53.66 - 69.87 | 64.07 | GZZY, HBWH, HBMC | 3 | |
| class 5 | 69.87 - 86.08 | 82.78 | HBLT, HBXG, GXHZ | 3 | |
|  |  |  |  |  | |
| Two-year-old pseudobulbs weight (2018) | | | | |  |
| class 1 | 12.58 - 45.33 | 26.01 | GXLY, SXXX, HBWF (*B. ochracea*), HBSY, SCLS, SYZS | 6 | |
| class 2 | 45.33 - 78.09 | 60.74 | HNSZ (*B. ochracea*), HBJM, YNBS, XSSC, SXZP (*B. ochracea*), HBJS, HBXS, HNSZ, HNCL, HBHF, SCLS (*B. ochracea*), HBXE | 12 | |
| class 3 | 78.09 - 110.84 | 94.05 | HBYL, YCWZ, HNLS (*B. ochracea*), HNLS, HBLC, HBMC | 6 | |
| class 4 | 110.84 - 143.60 | 124.67 | HBWH, JSNJ, SCWY, HBSN | 4 | |
| class 5 | 143.60 - 176.35 | 160.69 | GZZY, AHBZ, HBLT, HBXG, GXHZ | 5 | |
|  |  |  |  |  | |
| Three-year-old pseudobulbs weight (2019) | | | | |  |
| class 1 | 23.13 - 84.39 | 52.84 | GXLY, SXXX, HBWF (*B. ochracea*), HBSY, SYZS, HNCL, SCLS, XSSC | 8 | |
| class 2 | 84.39 - 145.65 | 111.09 | SCLS (*B. ochracea*), HNSZ (*B. ochracea*), HBXS, HBXE, HBJS, HBJM, YNBS, SCWY, SXZP (*B. ochracea*), HNSZ, HBHF, HNLS, HBLC, HBYL | 14 | |
| class 3 | 145.65 - 206.90 | 184.76 | HNLS (*B. ochracea*), JSNJ, HBMC, HBWH, YCWZ | 5 | |
| class 4 | 206.90 - 268.16 | 258.89 | GZZY, HBSN | 2 | |
| class 5 | 268.16 - 329.42 | 305.30 | HBLT, HBXG, AHBZ, GXHZ | 4 | |
|  |  |  |  |  | |
| Four-year-old pseudobulbs weight (2020) | | | | |  |
| class 1 | 31.63 - 113.68 | 68.81 | GXLY, HNCL, SXXX, HBSY, HBWF (*B. ochracea*), SCWY, SCLS, SYZS, HBXE, XSSC | 10 | |
| class 2 | 113.68 - 196.09 | 151.50 | HBJM, SCLS (*B. ochracea*), HNSZ (*B. ochracea*), HBXS, HBJS, HBLC, SXZP (*B. ochracea*), YNBS, HNSZ, HBYL | 10 | |
| class 3 | 196.09 - 278.32 | 224.06 | HNLS (*B. ochracea*), JSNJ, HNLS, HBHF, HBMC, YCWZ | 6 | |
| class 4 | 278.32 - 360.54 | 300.77 | GZZY, GXHZ, HBXG | 3 | |
| class 5 | 360.54 - 442.77 | 402.71 | HBSN, HBWH, AHBZ, HBLT | 4 | |
|  |  |  |  |  | |
| sum of one-, two-, three- and four-year-old pseudobulbs weight | | | | |  |
| class 1 | 72.27 - 251.03 | 154.10 | GXLY, SXXX, HBWF (*B. ochracea*), HBSY, SCLS, HNCL, SYZS | 7 | |
| class 2 | 251.03 - 429.70 | 318.60 | XSSC, HNSZ (*B. ochracea*), HBJM, HBXS, HBXE, SCLS (*B. ochracea*), HBJS, SCWY, YNBS, SXZP (*B. ochracea*), HNSZ | 11 | |
| class 3 | 429.70 - 608.36 | 490.57 | HBLC, HBYL, HBHF, HNLS (*B. ochracea*), HNLS, JSNJ, YCWZ | 7 | |
| class 4 | 608.36 - 787.03 | 714.61 | HBMC, GZZY, HBWH | 3 | |
| class 5 | 787.03 - 965.69 | 885.82 | HBSN, GXHZ, HBXG, AHBZ, HBLT | 5 | |

**Table S4 Ranking and identification of promising population based on pseudobulb growth ratio**

| **Class** | **Range** | **Mean** | **Populations** | **Number** |
| --- | --- | --- | --- | --- |
| One-year pseudobulb weight growth ratio | | | | |
| class 1 | 1.60 - 2.22 | 1.91 | HBMC, SCLS, SXXX, HBHF, HBWF (*B. ochracea*), SYZS, HBWH, HNCL, HBXG, HBJS, YCWZ, GXHZ, HBLT, HBYL, HBSY, HNLS | 16 |
| class 2 | 2.22 - 2.84 | 2.60 | GXLY, SCLS (*B. ochracea*), GZZY, SXZP (*B. ochracea*), HBJM, HBLC, YNBS | 7 |
| class 3 | 2.84 - 3.46 | 3.11 | JSNJ, HNSZ (*B. ochracea*), HBXE, AHBZ, XSSC | 5 |
| class 4 | 3.46 - 4.07 | 3.64 | HBSN | 1 |
| class 5 | 4.07 - 4.69 | 4.45 | HBXS, HNSZ, HNLS (*B. ochracea*), SCWY | 4 |
|  |  |  |  |  |
| Two-year pseudobulb weight growth ratio | | | | |
| class 1 | 1.82 - 3.14 | 2.75 | HNCL, SYZS, HBMC, HNLS, HBWH, SCLS (*B. ochracea*), HBJS | 7 |
| class 2 | 3.14 - 4.47 | 3.76 | SCLS, HBHF, HBSY, HBLC, HBXG, HBLT, HBYL, GXHZ, HBXE, SXXX, HBWF (*B. ochracea*), SCWY, GZZY, JSNJ | 14 |
| class 3 | 4.47 - 5.80 | 5.10 | GXLY, YCWZ, SXZP (*B. ochracea*), XSSC, HBJM, YNBS | 6 |
| class 4 | 5.80 - 7.13 | 6.20 | HNSZ (*B. ochracea*), HBXS, AHBZ | 3 |
| class 5 | 7.13 - 8.46 | 7.93 | HBSN, HNLS (*B. ochracea*), HNSZ | 3 |
|  |  |  |  |  |
| Three-year pseudobulb weight growth ratio | | | | |
| class 1 | 1.01 - 3.21 | 1.92 | HNCL, SCWY | 2 |
| class 2 | 3.21 - 5.41 | 4.36 | GXHZ, HBMC, SCLS, HBLC, HBXG, HBSY, SYZS, SXXX, SCLS (*B. ochracea*), HBXE, GZZY, HNLS, JSNJ, HBJS, HBYL, HBHF | 16 |
| class 3 | 5.41 - 7.61 | 6.34 | HBWF (*B. ochracea*), HBLT, HBWH, HBJM, GXLY, YCWZ, XSSC, SXZP (*B. ochracea*) | 8 |
| class 4 | 7.61 - 9.81 | 8.87 | HNSZ (*B. ochracea*), HBXS, AHBZ, YNBS, HNLS (*B. ochracea*) | 5 |
| class 5 | 9.81 - 12.00 | 11.07 | HBSN, HNSZ | 2 |

**Table S5 Ranking and identification of promising population based on polysaccharide content**

| **Class** | **Range** | **Mean** | **Populations** | **Number** |
| --- | --- | --- | --- | --- |
| Polysaccharide content of one-year-old pseudobulbs (2017) | | | | |
| class 1 | 18.84 - 26.88 | 18.84 | YNBS | 1 |
| class 2 | 26.88 - 34.92 | 33.76 | HNSZ (*B. ochracea*), HNLS (*B. ochracea*), HBJM | 3 |
| class 3 | 34.92 - 42.96 | 38.99 | SYZS, GXLY, YCWZ, HBSY, SXXX, HNSZ, HBXE, HBJS | 8 |
| class 4 | 42.96 - 51.00 | 47.24 | HBLC, HBXS, GXHZ, XSSC, HNLS, HBMC, HBWF (*B. ochracea*), HNCL, GZZY, JSNJ, HBSN, HBWH, SCLS | 13 |
| class 5 | 51.00 - 59.03 | 54.97 | HBHF, SCLS (*B. ochracea*), SCWY, HBLT, HBXG, AHBZ, HBYL, SXZP (*B. ochracea*) | 8 |
| Polysaccharide content of two-year-old pseudobulbs (2018) | | | | |
| class 1 | 15.91 - 24.69 | 15.91 | YNBS | 1 |
| class 2 | 24.69 - 33.46 | - | - | 0 |
| class 3 | 33.46 - 42.23 | 38.53 | HBJM, HNLS (*B. ochracea*), SYZS, HBSY, GXLY, HNSZ (*B. ochracea*), HBLC, YCWZ, HBJS, HBMC, HBXE | 11 |
| class 4 | 42.23 - 51.00 | 48.49 | HNSZ, GZZY, SXXX, HBXG, HNCL, XSSC, HBXS, AHBZ, JSNJ, SCLS, SCWY, GXHZ, HBSN | 13 |
| class 5 | 51.00 - 59.77 | 54.60 | HNLS, HBHF, HBWH, SCLS (*B. ochracea*), SXZP (*B. ochracea*), HBLT, HBWF (*B. ochracea*), HBYL | 8 |
| Polysaccharide content of three-year-old pseudobulbs (2019) | | | | |
| class 1 | 12.97 - 22.18 | 12.97 | YNBS | 1 |
| class 2 | 22.18 - 31.39 | 30.27 | HNSZ (*B. ochracea*), HNLS (*B. ochracea*) | 2 |
| class 3 | 31.39 - 40.60 | 36.97 | HBJM, GXLY, HBJS | 3 |
| class 4 | 40.60 - 49.81 | 45.35 | HBSY, YCWZ, HBXE, SYZS, HBSN, HNCL, HBLC, GZZY, HBMC, HNSZ, SXXX | 11 |
| class 5 | 49.81 - 59.02 | 54.11 | XSSC, SCLS (*B. ochracea*), HBLT, SCLS, GXHZ, JSNJ, HBXS, SCWY, HBWF (*B. ochracea*), HBYL, HNLS, HBXG, HBWH, HBHF, SXZP (*B. ochracea*), AHBZ | 16 |
| Polysaccharide content of four-year-old pseudobulbs (2020) | | | | |
| class 1 | 8.52 - 17.86 | 8.52 | YNBS | 1 |
| class 2 | 17.86 - 27.20 | 23.98 | GXLY, HNSZ (*B. ochracea*), HBJM, SXXX | 4 |
| class 3 | 27.20 - 36.54 | 31.32 | HNLS (*B. ochracea*), HBJS | 2 |
| class 4 | 36.54 - 45.89 | 40.43 | SYZS, HBXE, HBLC, HNSZ, YCWZ, HBMC, HBSY, SCLS (*B. ochracea*) | 8 |
| class 5 | 45.89 - 55.23 | 51.29 | GZZY, HBSN, XSSC, GXHZ, HNCL, SCWY, HNLS, HBWH, HBLT, SCLS, SXZP (*B. ochracea*), AHBZ, HBYL, HBHF, HBXG, HBXS, JSNJ, HBWF (*B. ochracea*) | 18 |
| Average of polysaccharide content of one-, two-, three- and four-year-old pseudobulbs | | | | |
| class 1 | 14.06 - 22.58 | 14.06 | YNBS | 1 |
| class 2 | 22.58 - 31.09 | 30.65 | HNSZ (*B. ochracea*) | 1 |
| class 3 | 31.09 - 39.61 | 35.68 | HBJM, HNLS (*B. ochracea*), GXLY, SYZS, HBJS, YCWZ | 6 |
| class 4 | 39.61 - 48.12 | 44.30 | HBSY, HBXE, SXXX, HBLC, HNSZ, HBMC, GZZY, XSSC, HNCL, HBSN | 10 |
| class 5 | 48.12 - 56.63 | 52.47 | GXHZ, HBXS, SCLS (*B. ochracea*), SCLS, JSNJ, HNLS, SCWY, HBWH, HBXG, HBLT, HBWF (*B. ochracea*), HBHF, AHBZ, SXZP (*B. ochracea*), HBYL | 15 |

**Table S6 Ranking and identification of promising population based on total phenols content**

| **Class** | **Range** | **Mean** | **Populations** | **Number** |
| --- | --- | --- | --- | --- |
| Total phenols content of one-year-old pseudobulbs (2017) | | | | |
| class 1 | 1.63 - 2.26 | 1.89 | HBJM, YNBS | 2 |
| class 2 | 2.26 - 2.88 | 2.63 | SXXX, YCWZ, HNLS (*B. ochracea*) | 3 |
| class 3 | 2.88 - 3.51 | 3.11 | HBSY, SYZS, GXLY, HBJS, HNSZ (*B. ochracea*) | 5 |
| class 4 | 3.51 - 4.14 | 3.84 | HNLS, SCLS, HBLC, XSSC, HBXS, HBXE, HNCL, HNSZ, GXHZ, GZZY, HBSN | 11 |
| class 5 | 4.14 - 4.77 | 4.36 | HBWF (*B. ochracea*), JSNJ, HBXG, SCLS (*B. ochracea*), HBWH, AHBZ, HBLT, HBMC, HBHF, SCWY, SXZP (*B. ochracea*), HBYL | 12 |
| Total phenols content of two-year-old pseudobulbs (2018) | | | | |
| class 1 | 1.41 - 2.11 | 1.61 | YNBS, HBJM | 2 |
| class 2 | 2.11 - 2.81 | 2.59 | HNLS (*B. ochracea*), HBSY, SXXX, HBWF (*B. ochracea*) | 4 |
| class 3 | 2.81 - 3.52 | 3.21 | SYZS, YCWZ, GXLY, HBJS, HBMC | 5 |
| class 4 | 3.52 - 4.22 | 3.81 | XSSC, HBXE, HNSZ, SCLS, HBWH, HNSZ (*B. ochracea*), HBLC, GZZY, HBXS, HNCL, HBXG, HNLS, SCWY | 13 |
| class 5 | 4.22 - 4.92 | 4.51 | HBSN, AHBZ, SCLS (*B. ochracea*), JSNJ, GXHZ, HBHF, SXZP (*B. ochracea*), HBLT, HBYL | 9 |
| Total phenols content of three-year-old pseudobulbs (2019) | | | | |
| class 1 | 0.90 - 1.70 | 1.11 | YNBS, HBJM | 2 |
| class 2 | 1.70 - 2.50 | - | - | 0 |
| class 3 | 2.50 - 3.30 | 2.91 | HNLS (*B. ochracea*), YCWZ, SXXX, HBSY, HBJS | 5 |
| class 4 | 3.30 - 4.10 | 3.86 | GXLY, HNCL, SYZS, HBXE, HBWH, GXHZ, SCLS, XSSC, GZZY, SXZP (*B. ochracea*), HBSN, AHBZ, HNSZ (*B. ochracea*), HBHF, HBLT | 15 |
| class 5 | 4.10 - 4.90 | 4.51 | SCLS (*B. ochracea*), HNLS, HBYL, SCWY, HBLC, HNSZ, HBWF (*B. ochracea*), HBXG, HBXS, HBMC, JSNJ | 11 |
| Total phenols content of four-year-old pseudobulbs (2020) | | | | |
| class 1 | 1.21 - 1.97 | 1.39 | HBJM, YNBS, GXLY, SXXX | 4 |
| class 2 | 1.97 - 2.73 | 2.24 | HBMC, HNLS (*B. ochracea*), YCWZ, HBJS | 4 |
| class 3 | 2.73 - 3.49 | 3.28 | HNSZ (*B. ochracea*), XSSC, SYZS | 3 |
| class 4 | 3.49 - 4.24 | 3.88 | HBXE, HBHF, SXZP (*B. ochracea*), HBSY, SCLS (*B. ochracea*), HNLS, GZZY, HBLC, HNCL, SCLS, HBYL, HNSZ, SCWY, GXHZ, HBXS, HBWF (*B. ochracea*), HBLT | 17 |
| class 5 | 4.24 - 5.00 | 4.50 | AHBZ, HBWH, HBSN, HBXG, JSNJ | 5 |
| Average of total phenols content of one-, two-, three- and four-year-old pseudobulbs | | | | |
| class 1 | 1.43 - 2.07 | 1.46 | YNBS, HBJM | 2 |
| class 2 | 2.07 - 2.71 | 2.57 | SXXX, HNLS (*B. ochracea*), YCWZ, GXLY | 4 |
| class 3 | 2.71 - 3.35 | 3.13 | HBSY, HBJS, SYZS | 3 |
| class 4 | 3.35 - 3.99 | 3.79 | HNSZ (*B. ochracea*), XSSC, HBXE, HBMC, SCLS, HNCL, HBWF (*B. ochracea*), HNLS, GZZY, HBLC | 10 |
| class 5 | 3.99 - 4.64 | 4.22 | HNSZ, HBWH, SCLS (*B. ochracea*), GXHZ, HBXS, HBHF, HBSN, SXZP (*B. ochracea*), AHBZ, SCWY, HBXG, HBLT, HBYL, JSNJ | 14 |

**Table S7 Ranking and identification of promising population based on militerane content**

| **Class** | **Range** | **Mean** | **Populations** | **Number** |
| --- | --- | --- | --- | --- |
| Militerane content of one-year-old pseudobulbs (2017) | | | | |
| class 1 | 0.51 - 1.20 | 0.91 | HNLS, HBXE, SXZP (*B. ochracea*), HBHF, SYZS, HBXG, HNLS (*B. ochracea*), YNBS, SCLS, HNCL, HBMC, HBXS | 12 |
| class 2 | 1.20 - 1.88 | 1.43 | HBJM, HNSZ (*B. ochracea*), HBSY, HBJS, SCWY, HBLT, GXHZ, GZZY, HBYL, HBWF (*B. ochracea*), XSSC, AHBZ, HBWH, HBSN, JSNJ | 15 |
| class 3 | 1.88 - 2.57 | 2.05 | GXLY, SXXX, HBLC, YCWZ | 4 |
| class 4 | 2.57 - 3.26 | 2.95 | SCLS (*B. ochracea*) | 1 |
| class 5 | 3.26 - 3.94 | 3.94 | HNSZ | 1 |
| Militerane content of two-year-old pseudobulbs (2018) | | | | |
| class 1 | 0.57 - 1.19 | 0.94 | SXZP (*B. ochracea*), HNCL, HBXG, HBMC, GZZY, HBHF, HBJS, SYZS, GXHZ, HBJM, HNSZ (*B. ochracea*), HBLC, HBXE | 13 |
| class 2 | 1.19 - 1.80 | 1.39 | YNBS, HNLS (*B. ochracea*), HBLT, SCLS, HBXS, HNLS, HBSN, HBYL, JSNJ, XSSC, HBSY, SCWY, AHBZ, HBWH | 14 |
| class 3 | 1.80 - 2.42 | 1.91 | YCWZ, SXXX | 2 |
| class 4 | 2.42 - 3.03 | 2.64 | HBWF (*B. ochracea*), GXLY, SCLS (*B. ochracea*) | 3 |
| class 5 | 3.03 - 3.65 | 3.65 | HNSZ | 1 |
| Militerane content of three-year-old pseudobulbs (2019) | | | | |
| class 1 | 0.60 - 1.09 | 0.87 | HBHF, HBMC, HNCL, HBXE, HBXG, GXLY, HBJM, HNLS, HNSZ (*B. ochracea*) | 9 |
| class 2 | 1.09 - 1.59 | 1.39 | HBXS, HBLC, HNLS (*B. ochracea*), GZZY, SYZS, SXZP (*B. ochracea*), HBJS, HBLT, HBSY, HBSN, HBWH, AHBZ, XSSC | 13 |
| class 3 | 1.59 - 2.08 | 1.85 | GXHZ, JSNJ, SCWY, SCLS, SXXX, YCWZ, HBYL | 7 |
| class 4 | 2.08 - 2.58 | 2.42 | YNBS | 1 |
| class 5 | 2.58 - 3.07 | 2.88 | HBWF (*B. ochracea*), SCLS (*B. ochracea*), HNSZ | 3 |
| Militerane content of four-year-old pseudobulbs (2020) | | | | |
| class 1 | 0.33 - 1.09 | 0.89 | HBYL, HBJS, HBXG, SXZP (*B. ochracea*), HBMC, HNSZ (*B. ochracea*), GXHZ, SYZS, SCLS | 9 |
| class 2 | 1.09 - 1.85 | 1.46 | GZZY, HNCL, HBXE, HBHF, HNLS (*B. ochracea*), HBSN, HBJM, HBXS, HBSY, AHBZ, HBLT, XSSC, SCWY, HBWH, YNBS, GXLY, HBWF (*B. ochracea*) | 17 |
| class 3 | 1.85 - 2.60 | 2.13 | YCWZ, SXXX, JSNJ, HNLS | 4 |
| class 4 | 2.60 - 3.36 | 3.06 | HBLC, SCLS (*B. ochracea*) | 2 |
| class 5 | 3.36 - 4.12 | 4.12 | HNSZ | 1 |
| Average of militerane content of one-, two-, three- and four-year-old pseudobulbs | | | | |
| class 1 | 0.86 - 1.43 | 1.13 | SXZP (*B. ochracea*), HBXG, HBMC, HNCL, HBHF, HBXE, SYZS, HBJS, HNSZ (*B. ochracea*), HNLS (*B. ochracea*), GZZY, HBJM, GXHZ, HBXS, HBYL, HNLS, SCLS, HBLT, HBSY | 19 |
| class 2 | 1.43 - 1.99 | 1.69 | HBSN, XSSC, SCWY, YNBS, AHBZ, HBWH, JSNJ, GXLY, HBLC, SXXX | 10 |
| class 3 | 1.99 - 2.56 | 2.04 | YCWZ, HBWF (*B. ochracea*) | 2 |
| class 4 | 2.56 - 3.13 | 2.96 | SCLS (*B. ochracea*) | 1 |
| class 5 | 3.13 - 3.69 | 3.69 | HNSZ | 1 |

**Table S8 Ranking and identification of promising population based on GRA**

| **Class** | **Range** | **Mean** | **Populations** | **Number** |
| --- | --- | --- | --- | --- |
| class 1 | 0.351 - 0.406 | 0.385 | YNBS, HBJM, GXLY, SYZS, SXXX | 5 |
| class 2 | 0.406 - 0.460 | 0.430 | HBSY, HBJS, HNCL, HNSZ (*B. ochracea*), HBXE | 5 |
| class 3 | 0.460 - 0.515 | 0.488 | SCLS, HNLS (*B. ochracea*), HBMC, YCWZ, XSSC, HBWF (*B. ochracea*), HBHF, HBLC, HNLS | 9 |
| class 4 | 0.515 - 0.570 | 0.543 | SXZP (*B. ochracea*), HBXS, GZZY, SCWY, HBYL, HBXG, SCLS (*B. ochracea*), HBWH, GXHZ | 9 |
| class 5 | 0.570 - 0.625 | 0.597 | JSNJ, HBSN, HBLT, AHBZ, HNSZ | 5 |

**Fig. S1 Hierarchical clustering analysis of pseudobulb weight, pseudobulb growth ratio and main active ingredient content**


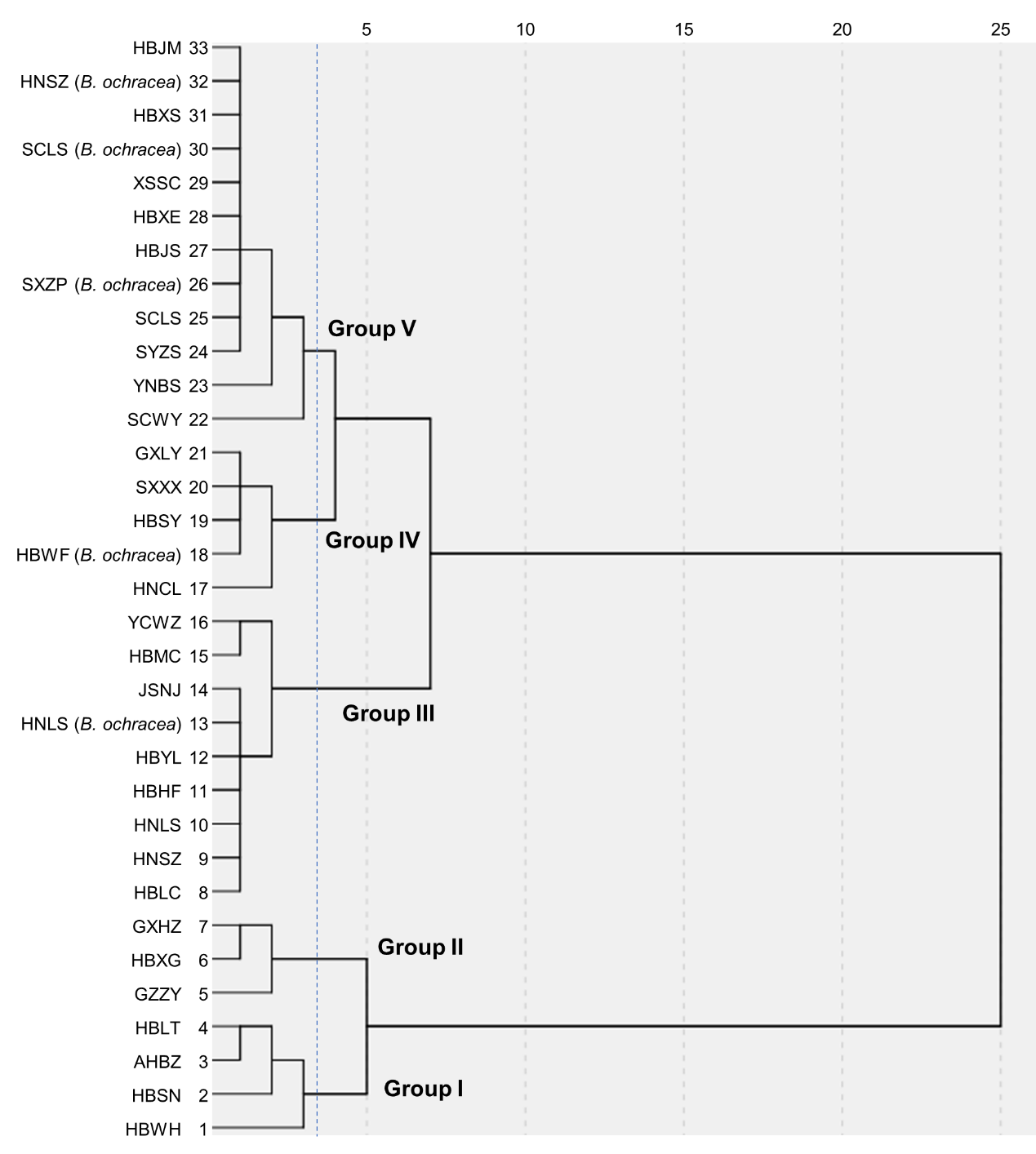

Supplement: Supplementary file 3 — Additional file 3: Table S3 Ranking and identification of promising population based on pseudobulbs weight. Table S4. Ranking and identification of promising population based on pseudobulb growth ratio. Table S5. Ranking and identification of promising population based on polysaccharide content. Table S6. Ranking and identification of promising population based on total phenols content. Table S7. Ranking and identification of promising population based on militerane content. Table S8. Ranking and identification of promising population based on GRA. Fig. S1. Hierarchical clustering analysis of pseudobulb weight, pseudobulb growth ratio and main active ingredient content. [file 12870_2022_3540_MOESM3_ESM.docx]
